# Implication of SARS-CoV-2 Immune Escape Spike Variants on Secondary and Vaccine Breakthrough Infections

**DOI:** 10.3389/fimmu.2021.742167

**Published:** 2021-11-03

**Authors:** Liyana Ahmad

**Affiliations:** Pengiran Anak Puteri Rashidah Sa'adatul Bolkiah (PAPRSB) Institute of Health Sciences, Universiti Brunei Darussalam, Bandar Seri Begawan, Brunei

**Keywords:** spike protein, SARS-CoV-2, secondary infection, breakthrough infection, vaccine, COVID-19, variants, immune evasion

## Abstract

COVID-19 pandemic remains an on-going global health and economic threat that has amassed millions of deaths. Severe acute respiratory syndrome coronavirus 2 (SARS-CoV-2) is the etiological agent of this disease and is constantly under evolutionary pressures that drive the modification of its genome which may represent a threat to the efficacy of current COVID-19 vaccines available. This article highlights the pressures that facilitate the rise of new SARS-CoV-2 variants and the key mutations of the viral spike protein – L452R, E484K, N501Y and D614G– that promote immune escape mechanism and warrant a cautionary point for clinical and public health responses in terms of re-infection, vaccine breakthrough infection and therapeutic values.

## Introduction

Since the outbreak of the COVID-19 pandemic in late 2019, SARS-CoV-2 virus has caused significant mortality and morbidity worldwide. While some regions of the world are seeing a dwindling of infected cases, others, especially densely populated regions are still recording high levels of infection. Efficient control of viral spread and decrease in mortality has been attributed to rigorous public health measures and global mass immunisation campaigns that have begun in December 2019. Manifestations of COVID-19 may range from mild to severe and life-threatening. While the majority of cases are subclinical, a significant portion of COVID-19 patients develop severe or fatal illness, which is often characterised by acute respiratory distress syndrome and hyper-inflammatory immune responses (1, 2). Epidemiological and public health surveillance efforts utilise viral genome information to track and monitor the growth of the pandemic. There is a growing concern for emerging viral strains that can potentially hamper public health strategies, post-vaccine or natural immunity and antiviral treatments. The World Health Organisation (WHO) has classified emerging variants with reference to the original Wuhan strain into different categories according to its hazard grade, with Variants of Concern (VOCs) as the ones needing urgent monitoring of. Currently, there are four VOCs which include Alpha (B.1.1.7), Beta (B.1351), Gamma (P.1) and Delta (B.1.617.2) variants that are at the front of the pandemic wave, with the latter seemingly outperforming the others. In response to this, unprecedented scale of sequencing and tracking of SARS-CoV-2 genome evolution has been initiated to accommodate the rapid accumulation of viral mutations and to understand the evolutionary adaptation of the virus in humans in hope to better design effective COVID-19 vaccines and treatment options.

## Evolutionary Pressure Driving the Emergence of SARS-CoV-2 Variants

SARS-CoV-2 virus is a large RNA virus with a 30-kb genome that encodes for four structural proteins: spike glycoprotein, nucleocapsid, membrane and envelope proteins (3). The genome of SARS-CoV-2 has been reported to accumulate two nucleotide substitutions per month, which is relatively slow for an RNA virus owing to its proofreading 3’–5’exoribonuclease (4). While most chance mutations are often silent that lead to no changes at biological level or deleterious that compromise viral fitness, some may confer selective advantage for its fitness. This leads to their propagation in subsequent viral populations, which carry advantageous phenotypes and are often subjected to a purifying selection. This is evident by a genetic drift reported in SARS-CoV-2 variants particularly in their spike and nucleocapsid gene sequences which are most variable (5). Additionally, evidence of independently co-occurring or converging mutations in SARS-CoV-2 genome also suggests that there exists a persistent and increasing selection pressure on the virus, which can occur both at population and individual patient levels.

In an infected individual, variants can independently inhabit different tissues and up to four variants have reportedly been identified within a patient (6, 7). However, it is unlikely that minor intra-host mutations will propagate and become fixed due to their low frequencies and natural bottleneck effects (8, 9). While minor viral genetic mutations within infected individuals are expected, a genetic drift may raise concern at the population level. Currently, the pandemic has already infected over 200 million cases globally, leaving behind convalescent individuals with natural immunity against the virus. Additionally, as immunisation has begun globally, a growing portion of the population is now carrying vaccine-induced immunity against the circulating virus. The increasing degree of immunity in the human population is inevitably conferring a great deal of selective pressure on the virus that promotes the rise of antibody escape mutants. The emergence of immune escape mutants is perhaps most apparent in chronic COVID-19 patients as documented in multiple reports. Persistent infections with SARS-CoV-2 seen in immunocompromised individuals who cannot effectively fight infection accelerates viral evolution which gives rise to large genomic diversity and mutations in the viral spike, ORF1ab, ORF8 and nsp1 proteins (10, 11). These mutations were observed to recur and independently emerge in these patients (10, 11).

In addition to vaccination, immunotherapies have also been used as COVID-19 disease interventions. These include repurposed drugs such as antiviral remdesivir and corticosteroids, convalescent plasma therapy that carry neutralising antibodies, and antiviral monoclonal antibodies (12, 13). Unfortunately, these may also pose as drivers for advantageous mutations. Reports on the use of convalescent plasma has shown to contribute to the generation of antibody escape variants (14–16). The use of sub-optimal antibodies in convalescent therapy and re-infection at the face of a decaying or a partial primary immunity may provide a selective pressure for immune escape mutations.

### Spike Variants Pose a Threat to Current Vaccines and Therapeutics

**Figure 1:**
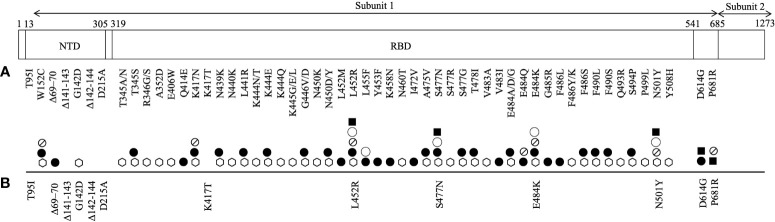
An illustration of SARS-CoV-2 spike protein mutations identified in its subunit 1 that have been **(A)** shown to promote resistance to neutralizing monoclonal antibodies (⎔) and serum antibodies (from convalescent (●) and/or vaccinated (⦰) individuals), and increase the protein affinity to ACE2 receptor (◯) and viral infectivity (■) and **(B)** reported in breakthrough and/or secondary infections.

Spike protein of SARS-CoV-2 defines the tropism of the virus, facilitates its spread, and modulates host immune function. It is the viral entry point of the host cell by which it binds to host angiotensin-converting enzyme 2 (ACE2) receptor. It is composed of 1273 amino acids and harbours two subunits that are bridged by a furin cleavage site. Subunit 1 contains two important domains: N terminal domain (NTD) and receptor binding domain (RBD) (**Figure 1**). It is also the main target of vaccination efforts as the majority of serum neutralising activity is directed on its RBD (17, 18).

A primary infection with SARS-CoV-2 provokes immune responses that generate potent anti-SARS-CoV-2 neutralising antibodies and CD4^+^ and CD8^+^ T cell responses which clear the infection and culminate with an immune response memory to fight future infection. As the pandemic continues to sweep across the globe, it has gradually become apparent that a primary infection with SARS-CoV-2 does not provide a full protection. Although rare, cases of re-infection were documented as early as August 2020 and many are expected to be underreported as it can be difficult to distinguish between re-infection and prolonged viral shedding (19–22). Additionally, a comprehensive whole genome sequencing is necessary to identify two separate episodes of infection. A natural exposure to SARS-CoV-2 generate anti-SARS-CoV-2 IgG antibodies that are largely protective for at least 6 months post-infection (23, 24). Repeat infection can be caused by a low potency and quality of immune memory, or a decline in anti-SARS-CoV-2 antibody titre over time. However, a report of a re-infection case indicates that a secondary infection with SARS-CoV-2 occurred in the presence of intact anti-SARS-CoV-2 antibody titre, suggesting that a seropositivity does not necessarily guarantee immunity and raising concern about immune escape variants that can overcome previous immunity (25).

Reports on vaccine breakthrough infections in vaccinated individuals are also accumulating, raising concern of developing escape mutants in the face of immune selection (26–30). A breakthrough infection is defined as a detection of SARS-CoV-2 antigen or RNA more than 14 days after receiving a COVID-19 vaccine. To date, over 10, 000 breakthrough infections have been reported (31). The durability of vaccine-induced antibodies is yet to be determined, but vaccines against SARS-CoV-2 elicit more robust antibody responses than a natural infection which may suggest a more stable protection (32). Nevertheless, both natural- and vaccine-induced anti-SARS-CoV-2 antibodies demonstrate comparable neutralising power (33).

As of June 14^th^, a total of 6,238 nucleotide substitutions, 56 insertions and 278 deletions in the spike protein have been recorded in clinical isolates (34, 35). The most profound mark of SARS-CoV-2 evolution is perhaps the D614G mutation that emerged as early as 6 months into the outbreak. D614G mutation is located outside of the RBD (**Figure 1**). D614G variant bypassed natural bottlenecks due to its selective advantages that lead to improved viral infectivity and transmissibility and reduced viral sensitivity to neutralising convalescent sera (36–38). It immediately outcompeted other sequence groups and became ubiquitous worldwide. All VOCs that are driving the current pandemic waves harbour this dominant mutation.

The main target of serum neutralising antibodies is the RBD of the virus spike protein, which upon recognition prevents the virus from gaining entry to the host cell. A growing list of mutations in the RBD and NTD of the spike protein has been reported to resist neutralisation by therapeutic monoclonal antibodies, convalescent sera and vaccinee sera (**Figure 1**). Among them, a few merit concern as they have also recently been identified in re-infection and breakthrough infections with VOCs, likely for their location in the immune epitope region of the RBD that may impact antigenicity, hinder immune neutralising antibody binding and promote pathogenesis (**Table 1**) (17, 18).

**Table 1:** Mutations of SARS-CoV-2 spike protein, their phenotypic impact *in vitro, in vivo* or *in silico*, and reports in re-infection and vaccine breakthrough infection cases.

Spike mutations		G142D	L452R	E484K	N501Y	D614G	P681R	References
**Variants**		Delta	Delta	Alpha	Alpha	Ubiquitous	Delta	(35, 39)
			Kappa	Beta	Beta		Kappa	
			Epsilon	Gamma	Gamma			
			Iota	Zeta	Mu			
				Eta				
				Iota				
				Kappa				
				B.1.620				
				Mu				
**Phenotypic impact**	Resistance to neutralising mAb	Increases	Increases	Increases	Increases	–	–	(33, 36, 40–45)
	Resistance to convalescent sera	–	Increases	Increases	–	Increases	–	(37, 40, 42, 43, 46–48)
	Resistance to vaccine sera	–	Increases	Increases	Increases	–	–	(33, 43, 48–52)
	Affinity to ACE2 receptor	–	Increases	Increases	Increases	–	–	(53–55)
	Infectivity	–	Increases	–	Increases viral shedding in animal *in vivo*	Increases	Increases	(36, 38, 40, 48, 56–58)
**Reported in re-infection cases**		–	A case in Panama	Cases in Brazil	Cases in UK and Brazil	Cases in Brazil, Hong Kong, India, Panama, UK, USA	–	(19, 25, 59–63)
**Reported in vaccine breakthrough cases**		Cases in India	Cases in California	Cases in India, Israel, and New York	Cases in California, India and Israel	Cases in Israel and New York	Cases in India	(57, 64–67)

mAb, monoclonal antibodies.

E484K mutation first emerged in late 2020 and has now been gaining prevalence among the circulating strains. It has been acquired by at least 9 strains including VOCs Alpha, Beta and Gamma, and other variants under monitoring Zeta (P.2), Eta (B.1.525), Iota (B.1.526), Kappa (B.1.617.1), B.1.620 and Mu (B.1.621) (39). It is noteworthy to mention that five reinfection cases of E484K-carrying variants (Gamma and Zeta), which primary infections were of non-E484K variants, have been reported in Brazil as early as December 2020. Two of the cases reported the presence of anti-SARS-CoV-2 IgG antibodies during re-infection suggesting the possibility of E484K mutation in aiding antibody evasion (59). Furthermore, breakthrough infections in vaccinated individuals are also growing in number. A cohort study identified a slight reduction of protection by vaccines against subsequent infection with E484K-carrying Alpha and Beta variants of concern (64). Another report also documented an infection of a vaccinated individual with E484K-carrying variant (65). In another study, a full vaccination elicited a high level of neutralising antibodies that were capable of inhibiting an E484K variant *in vitro* but failed to pre-empt a high viral replication (66).

*In vitro* studies showed that E484K mutation can significantly reduce binding and resist neutralisation by convalescent and vaccine-induced sera and monoclonal antibodies (33, 40–42, 46, 47, 49–51). Additionally, *in silico* and cryo-EM studies have found that E484K mutation enhances the virus affinity for host ACE2 receptor binding which may account for the increased infectivity of these mutant variants (53, 54). Its impact on infectivity *in vitro* has been revealed in an infection assay of murine ACE2-expressing HEK29T cells with E484K-carrying pseudotyped viruses whereby 3-fold more infection was observed (68). The prevalence of E484K-carrying variants has been increasingly reported in viral isolates, presenting at low frequency in the circulating strain populations (42), likely due to the positive selection that provides for an immune escape and a greater transmissibility. This is consistent with *in vitro* evolutionary studies that showed that E484K mutation is readily introduced in the viral genome when cultured in the presence of anti-SARS-CoV-2 neutralising antibodies or ACE2 receptor (42, 47, 54). Furthermore, four E484 mutational changes at this residue position demonstrated an immune escape phenotype in presence of vaccinee sera, highlighting the importance of this residue as part of the dominant neutralising epitope (40).

Also gaining in frequency among circulating variant sequences is N501Y substitution that has been acquired by three VOCs Alpha, Beta and Gamma. A report has demonstrated that N501Y mutation reduces the virus sensitivity to neutralising monoclonal antibodies and vaccine-induced polyclonal antibodies (33, 51). *In vitro* infection assay of murine ACE2-expressing HEK293T cells with N501Y-carrying pseudotyped virus also showed that this mutation caused a 5-fold increase in viral infectivity (68). Additionally, it was shown that it is positively selected *in vitro* when the virus is grown in the presence of ACE2 to which it developed a higher binding affinity, and *in vivo* in a chronically infected immunocompromised patient (54, 69, 70). The increase in ACE2 tropism consequently promotes viral replication, transmission and shedding (69). N501Y-carrying variants (VOCs Alpha and Gamma) have been reported in four re-infection cases in which primary episode of infection agents were of non-N501Y-carrying variant (25, 59). Three of these cases described the presence of anti-SARS-CoV-2 IgG antibodies at the timepoint of secondary infection, raising speculation of antibody escape that may be mediated by this mutation. Furthermore, several independent reports of breakthrough infections with N501Y-caryying variant had been reported in vaccinated individuals suggesting an evasion of humoral immunity (65, 67).

While E484K only mildly increases the affinity of the RBD for ACE2 receptor, N501Y appears to substantially enhance it by allowing it to engage the receptor for longer (71–73). This is in agreement with an observation of pseudotyped viruses carrying N501Y mutation demonstrating a higher infectivity *in vitro* than those carrying E484K mutation (68). In contrast, the E484 residue in RBD engages more with antibodies than with ACE2 receptor, which may explain the relevance of E484K mutation in mediating antibody escape (72). The co-existence of these two mutations can produce a synergistic effect whereby they dramatically improve spike binding affinity to ACE2 receptor and reinforce the virus ability to evade immunity (68, 72, 73). The importance of these mutations for viral fitness is highlighted by their convergence in certain variants, including Beta, Gamma, and Mu (**Table 1**).

Another mutation that has gained attention is L452R substitution which can resist neutralisation by monoclonal antibodies and vaccinee and convalescent sera (36, 40, 43, 46, 48, 52). It provides a greater affinity of binding to ACE2 receptor and thus promotes viral replication and infectivity (48, 55, 56). A report of breakthrough infections in fully or partially vaccinated healthcare workers and a secondary infection with L452R-carrying variants have been described, highlighting the relevance of this mutation in mediating viral immune escape (60, 67). L425R mutation has been identified in the circulating VOC Delta and other variants including Epsilon (B.1.427/B.1.429), Iota and Kappa. Of important note, Delta variant is currently posing the most concern and threat to public health, as it has displaced other variants owing to its pronounced ability to transmit and escape antibodies (monoclonal antibodies and convalescent and vaccinee sera) (74–76). Increasing numbers of breakthrough infection with Delta variant have been reported (57, 77). Other mutations, in addition to L452R of the RBD, have been attributed to its explosive spread. Upstream NTD mutation G142D has been shown to resist binding by monoclonal antibodies which may be mediated by the change of the conformation of the spike protein that deters antibody binding (44, 45). Downstream P681R mutation in furin cleavage site promotes cleavage and processing of the spike protein and hence the entry efficiency and infectivity of the virus *in vitro* (57, 58).

Although current available vaccines are engineered based on the spike protein of the Wuhan reference strain, vaccinated individuals are expected to carry potent neutralising antibodies that are cross-reactive which can circumvent the issue of variants (78). Nevertheless, the growing evidence for immune evasion by emerging spike variants prompts a heavier consideration for a more adequate and effective vaccine design guided by genomic surveillance that can stimulate a more durable protection from SARS-CoV-2 infection.

## Research Gaps and Prospects

SARS-CoV-2 spike and nucleocapsid proteins are hotspots of genetic modification owing to the host immune selection pressure. Genomic surveillance is increasingly becoming a powerful tool in guiding public health responses and health interventions and therefore in controlling the trajectory of this pandemic. This will provide a wealth of databases that can be used to infer decisions on evaluating health interventions, vaccine efficacy, inform therapeutic development and design, assess the risk of re-infection and breakthrough infections, and tackle the issue of immune and diagnostic escape variants.

The growing relevance of a select spike mutations urges immediate investigation, particularly E484K, to discern whether it will become the next consensus sequence (as did D614G). Not explored in this article is the impact of spike or nucleocapsid mutations on primary diagnosis which have been documented elsewhere (79–81). Other clinical indicators should therefore accompany molecular-based COVID-19 diagnostic tests – more so in the wake of a growing trend of re-infection and breakthrough infection – to guide not only clinical evaluation, but also isolation and discharge decisions.

Because a waning and heterogeneous protective immunity has been suggested as risk factors of secondary and breakthrough infections, the identification of correlate of protection with a consideration of immune escape mutations, is crucial in identifying the most optimal vaccine design that promises a more preserved antibody titre and protection. One can expect the need for periodic reformulation of the vaccines in the future as ‘boosters’ to adapt to the most common variant at the time, recover the declining immunity and mitigate the risk of future emerging variants. Future interventions could consider harnessing other facet of protective immune responses, in addition to humoral immunity, to relieve selective pressure for immune escape variants. Notably, T cell-mediated immune responses are reported to be relatively more stable following SARS-CoV-2 infections and are potent at targeting the spike protein (82). Additionally, the heterogeneity of resistance profile of spike variants to monoclonal antibodies suggests that administering a cocktail of antibodies would be a more beneficial approach at targeting these variants (42). Other mutations outside of the spike gene should also be warranted surveillance in studying the evolution of SARS-CoV-2 and its interaction with human host to pre-empt potential novel variants that may affect current efforts of controlling the pandemic.

## Author Contributions

LA conducted the study and agrees to be accountable for the content of the work.

## Conflict of Interest

The author declares that the research was conducted in the absence of any commercial or financial relationships that could be construed as a potential conflict of interest.

## Publisher’s Note

All claims expressed in this article are solely those of the authors and do not necessarily represent those of their affiliated organizations, or those of the publisher, the editors and the reviewers. Any product that may be evaluated in this article, or claim that may be made by its manufacturer, is not guaranteed or endorsed by the publisher.
